# Preferential use of Siglec-1 or Siglec-10 by type 1 and type 2 PRRSV strains to infect PK15^S1–CD163^ and PK15^S10–CD163^ cells

**DOI:** 10.1186/s13567-018-0569-z

**Published:** 2018-07-18

**Authors:** Jiexiong Xie, Isaura Christiaens, Bo Yang, Ivan Trus, Bert Devriendt, Tingting Cui, Ruifang Wei, Hans J. Nauwynck

**Affiliations:** Department of Virology, Immunology and Parasitology, Faculty of Veterinary Medicine, Ghent University, Salisburylaan 133, 9820 Merelbeke, Belgium

## Abstract

Cellular entry mediators define whether the cell is permissive to PRRSV infection. Porcine sialoadhesin (pSn, Siglec-1) and CD163 are main entry mediators facilitating infection of porcine macrophages by PRRSV. Recently, Siglec-10 was demonstrated to be an alternative receptor for PRRSV. To examine if virulence and pathogenicity of PRRSV strains could be correlated with the use of different Siglecs, a PK15 cell line recombinantly expressing Siglec-1 and CD163 (PK15^S1–CD163^) and a PK15 cell line recombinantly expressing Siglec-10 and CD163 (PK15^S10–CD163^) were used to compare the virus replication of 7 genotype 1 subtype 1 strains (G1s1), 2 genotype 1 subtype 3 (G1s3) strains and 5 genotype 2 (G2) strains. Some strains (08VA (G1s1), 13V117 (G1s1), 17V035 (G1s1), VR2332 (G2)) were poor virus producers (<10^4^ TCID_50_/mL), while other strains (07V063 (G1s1), 13V091 (G1s1), Su1-Bel (G1s3), MN-184 (G2), Korea17 (G2) and SDSU-73 (G2)) easily grew up to ≥10^6^ TCID_50_/mL. PK15^S10–CD163^ cells exhibited a higher efficiency in virus production per infected cell than the PK15^S1–CD163^ cells. The G1s1 strains LV and 07V063 infected more cells in the PK15^S1–CD163^, whereas the 94V360 and 08VA strains preferred PK15^S10–CD163^. The highly virulent G1s3 strains Lena and Su1-Bel showed a strong preference for PK15^S1–CD163^. The G2 strains MN-184, SDSU-73, Korea17 had a much higher infection rate in PK15^S10–CD163^, while the reference strain VR2332 and the NADC30 strain had a slight preference for PK15^S1–CD163^. Differences in receptor use may influence the outcome of a PRRSV infection in pigs and explain in part the virulence/pathogenicity of PRRSV strains.

**Electronic supplementary material:**

The online version of this article (10.1186/s13567-018-0569-z) contains supplementary material, which is available to authorized users.

## Introduction

Porcine reproductive and respiratory syndrome virus (PRRSV) is a member of the Arterivirus, genus, family *Arteriviridae*, order *Nidovirales* [1] causing respiratory disorders in piglets and reproductive problems in adult animals. PRRSV infections cause major economic losses in the pig industry worldwide [2, 3]. In vivo, the virus infects a subpopulation of tissue macrophages, and also subpopulation of monocyte and bone marrow derived dendritic cells [4–9]. In vitro, efficient PRRSV replication is observed in primary porcine alveolar macrophages (PAM), differentiated monocytes [10] and for certain strains (mainly after adaptation) in African green monkey kidney derived cells, e.g. MARC-145 [11]. Porcine sialoadhesin (pSn, also known as Siglec-1) and porcine CD163 (pCD163) have been reported to be the main entry mediators for PRRSV [12–14]. In the classic PRRSV entry model, the virus binds to and is internalized into the macrophages via pSn through interacting with the viral GP5/M protein complex. Once inside the cell, pCD163 mediates the viral disassembly and genome release. However, recent studies demonstrated that PRRSV do not only infect sialoadhesin positive, but also sialoadhesin negative cells [15, 16]. Moreover, Siglec-1 knockout pigs are still susceptible to PRRSV [17]. These results indicated that PRRSV may use alternative entry mediators to infect the host. Indeed, we have recently demonstrated that Siglec-10, a sialic acid binding protein belonging to the same family as Siglec-1, is able to facilitate the infection of non-permissive cells by PRRSV [18]. It is very well possible that even more siglecs and/or siglec-like molecules exist. To analyze the receptor use of different PRRSV strains (7 G1s1, 2 G1s3 and 5 G2), a stably transfected cell line expressing both Siglec-10 and CD163 (PK15^S10–CD163^) was established and compared with the earlier developed cell line stably expressing both Siglec-1 and CD163 (PK15^S1–CD163^) [10].

## Materials and methods

### Cells and viruses

PK15 cells were cultivated in Dulbecco’s Modified Eagle Medium (D-MEM) supplemented with 10% fetal bovine serum (FBS), 100 U/mL penicillin, 0.1 mg/mL streptomycin. MARC-145 cells, PK15^S1–CD163^ and PK15^S10–CD163^ cells were cultivated in Modified Eagle Medium (MEM), supplemented with 10% FBS, 100 U/mL penicillin, 0.1 mg/mL streptomycin. The following PRRSV strains were analyzed in our study: LV (prototype G1s1, 13 passages in PAM), 94V360 (G1s1, 3 passages in PAM), 07V063 (G1s1, 3 passages in PAM), 08VA (G1s1, 4 passages in PAM), 13V091 (G1s1, 4 passages in PAM), 13V117 (G1s1, 3 passages in PAM), 17V035 (G1s1, 2 passages in PAM), Lena (G1s3, 4 passages in PAM), Su1-Bel (G1s3, 4 passages in PAM), VR-2332 (G2, 4 passages in MARC-145 and 2 passages in PAM), MN-184 (G2, 3 passages in MARC-145 and 3 passages in PAM), SDSU-73 (G2; 3 passages in MARC-145 and 3 passages in PAM), NADC30 (G2, 3 passages in MARC-145 and 3 passages in PAM) and Korean17 (3 passages in PAM). Information on the origin and virus characteristics are summarized in Table 1.

**Table 1:** **Information on the virus strains used in the study**

Strain	Year of isolation	Origin	Passages in MARC-145/PAM	Virulence score	Macrophage (mø) tropism	Genotype	Accession number	References
LV	1991	Netherlands	13 in PAM	1+	Sn^+^ mø	G1s1	M96262	[28]
94V360	1994	Belgium	3 in PAM	NA	NA	G1s1	JF304781	[31]
07V063	2007	Belgium	3 in PAM	3+	Sn^+^ and Sn^−^ mø	G1s1	GU737264	[16]
08VA	2008	Belgium	4 in PAM	0	Sn^+^ and Sn^−^ mø	G1s1	GU737266	[16]
13V091	2013	Belgium	4 in PAM	4+	Sn^+^ and Sn^−^ mø	G1s1	KT159248	[16]
13V117	2013	Belgium	3 in PAM	0	Sn^+^ and Sn^−^ mø	G1s1	KT159249	[16]
17V035	2017	Belgium	2 in PAM	0	NA	G1s1	This study	NA
Lena	2007	Belarus	4 in PAM	6+	Sn^+^ and Sn^−^ mø	G1s3	JF802085	[32]
SU1-Bel	2010	Belarus	4 in PAM	5+	NA	G1s3	KP889243	[33]
VR2332	1990	USA	4 in MARC-145 and 2 in PAM	2+	Sn^+^ and Sn^−^ mø	G2	U87392	[34]
SDSU-73	1995	USA	3 in MARC-145 and 3 in PAM	4+	NA	G2	JN654458	[28]
MN-184	2001	USA	3 in MARC-145 and 3 in PAM	5+	Sn^+^ and Sn^−^ mø	G2	EF488739	[28]
NADC-30	2008	USA	3 in MARC-145 and 3 in PAM	3+	NA	G2	JN654459	[28]
Korean 17	2017	Korean	3 in PAM	NA	NA	G2	This study	NA

NA: not available; S1: PK-15^S1–CD163^; S10: PK-15^S10–CD163^; G1s1: type1 subtype 1; G1s3: type1 subtype 3; G2: type 2

### Transfection and clone selection

**Figure 1:**
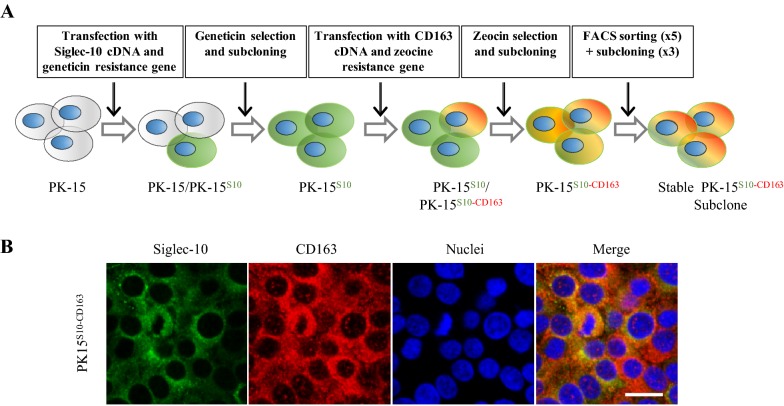
**Construction strategy and immunofluorescence staining of PK15**^**S10–CD163**^ **cells. A** Schematic representation of the construction of the PK15^S10–CD163^ cell line. To construct a cell line co-expressing Siglec-10 and CD163, PK15 cells were first transfected with a plasmid containing Siglec-10 cDNA and a geneticin resistance gene. The cells were subcloned and screened for Siglec-10 expressing cells. After selection for geneticin resistance, the obtained PK15^S10^ cells were transfected with a plasmid containing the CD163 cDNA and a zeocin resistance gene, which allowed the selection of cells expressing both Sn and CD163. **B** Immunofluorescence staining for PK15^S10–CD163^. The green color represents Siglec-10, while the red color represents CD163. The nuclei of the cells are blue (Hoechst). Scale bar: 25 µm.

PK15 cells were transfected in the presence of lipofectamine 3000 (Invitrogen) according to the manufacturer’s instructions. PK15 cells were first transfected with a plasmid containing the Siglec-10 cDNA and a geneticin resistance gene as described previously [18]. Afterwards, the obtained PK15^S10^ cells were transfected with a plasmid containing the CD163 cDNA and a zeocin resistance gene and single cell cloned. For the selection of PK15^S10–CD163^ cells, zeocin (200 μg/mL, Invitrogen) was used. Cells were further sorted and subcloned three times with a FACSAria III (Becton–Dickinson) to obtain stably expressing cell clones (Figure 1).

### Fluorescence activated cell sorting (Facs)

Cells were trypsinized with 0.075% trypsin (Sigma-Aldrich) and washed 2 times and harvested with MEM containing 1 mM EDTA and 1% FBS. The cells were labeled with anti-CD163 monoclonal antibodies (2A10, IgG_1_, AbD, serotec) or mAb against siglec-10 [18]. Isotype-matched monoclonal antibodies (13D12, directed against PRV gD) [19] were used as control. The incubation was performed in the presence of 1 mM EDTA and 1% FCS for 30 min on ice. AlexaFluor 647-conjugated polyclonal antibodies against mouse IgG_1_ (Invitrogen) were used as secondary antibodies. Incubation was performed in the presence of 1 mM EDTA and 1% FCS for 30 min on ice in the dark. FACS sorting was performed with a FACSAria III. Two million cells were used for each sorting, doublets were excluded with a gating strategy based on forward light scatter and sideward light scatter. Dead cells were excluded with propidium iodide staining. Sorted cells were collected in the RPMI/DMEM (1:1) supplemented with 30% FCS, 1% P/S and 0.5% gentamicin and directly used for a subcloning strategy. Subcloning was performed by limiting dilution as described for cloning of hydridomas [20]. The expression of Siglec-10 and CD163 in the subclones was checked by immunofluorescence.

### Immunofluorescence staining

Cells were fixed with methanol for single stainings. Primary monoclonal antibodies were used: mAb 1G10 against Siglec-10 (IgG_1_) [18]; mAb 41D3 against Siglec-1 [21] or mAb 2A10 against CD163 (IgG_1_, AbD, serotec). 13D12 was used as isotype control. As a secondary antibody, fluorescein isothiocyanate (FITC)-conjugated polyclonal goat anti-mouse IgG antibodies (Invitrogen) were used. The expression of Siglec-1, Siglec-10 and CD163 was examined with a fluorescence microscope (Leica Microsystems GmbH, Germany). For double staining of Siglec-10 and CD163, goat polyclonal antibodies against CD163 (R&D Systems, Minneapolis) and mAb 1G10 against Siglec-10 were incubated at 37 °C for 1 h. After three washes with PBS, cells were incubated with rabbit anti-goat AF594 for 1 h at 37 °C. After blocking with negative goat serum for 30 min at 37 °C cells were further incubated with FITC-labelled goat anti-mouse IgG. Cell nuclei were stained with Hoechst 33 342.

### Flow cytometry

PK15^S10–CD163^, PK15^S1–CD163^ and PK-15 cells were trypsinized with 0.075% trypsin (Sigma-Aldrich). 10^6^ cells of each cell line were collected for each experimental condition. Cells were washed two times with PBS. For intracellular staining, cells were fixed with 4% PF and permeabilized with 0.1% triton before incubation with primary antibodies. For surface staining, cells were directly incubated with the primary antibodies against CD163 (mouse IgG_1_, 2A10), Siglec-10 (mouse IgG_1_, 1G10), Sn (mouse IgG_1_, 41D3), PRV gD (isotype matched control mAb mouse IgG_1_, 13D12). AF647-conjugated goat anti-mouse IgG_1_ antibodies (Invitrogen) were used as secondary antibodies. The staining procedures were the same as described for Facs above. Flow cytometry was performed with Cytoflex (Beckman coulter). 10 000 events were recorded and 1000 events were displayed, doublets were excluded with a gating strategy based on forward light scatter and sideward light scatter.

### Analysis of PRRSV attachment, internalization, disassembly and infection in PK15^S1–CD163^ and PK15^S10–CD163^ cells by immunofluorescence

Cells were seeded at 2 × 10^5^ cells/mL (1 mL/well in 24-well plate) and after 3 days of cultivation, cells were inoculated with PAM grown LV at a multiplicity of infection (MOI) of 1. After 1 h of incubation at 4 °C, cells were fixed with methanol in order to assess virus attachment. To quantify the internalized particles, to analyze virus disassembly and virus infection, cells were fixed after 1, 5 and 24 h of incubation at + 37 °C with ice-cold methanol (−20 °C, 10 min). The virus was stained with mAb 13E2, directed against PRRSV nucleocapsid (IgG_1_, 1:50) and secondary FITC-conjugated polyclonal goat anti-mouse IgG antibodies (Invitrogen, Molecular Probes, Belgium). Virus particles and number of PRRSV infected cells were counted on images acquired with a TCS SPE confocal system (Leica Microsystems GmbH, Germany). Twenty randomly selected fields were taken for calculating the virus particles and percentage of infected cells.

### PRRSV infection kinetics in PK15^S1–CD163^ and PK15^S10–CD163^ cells

PK15^S10–CD163^ and PK15^S1–CD163^ cells were seeded at a concentration of 200 000 cells/mL in 24 well plates with glass inserts (1 mL/well). After 3 days of cultivation, cells were inoculated with 200 µL of a virus stock containing 10^5^ TCID_50_/mL of four different PRRSV strains grown on PAMs: LV, Lena, MN-184 and VR2332. After 0, 12, 24, 48 and 72 hours post-inoculation (hpi), supernatant was collected, centrifuged (10 min, 300 *g*, 4 °C) and stored at −70 °C prior to virus titration on PAM [6]. Cells on inserts were fixed with methanol at the indicated time points and stained for immunofluorescence analysis. The PRRSV N protein was stained with primary mAb 13E2 directed against PRRSV nucleocapsid (IgG_1,_ 1:50) and secondary FITC-conjugated polyclonal goat anti-mouse IgG antibodies (Invitrogen, Molecular Probes, Belgium). Virus positive cells were counted on images acquired with a TCS SPE confocal system (Leica Microsystems GmbH, Germany). Twenty images were taken for each experimental condition, and the percentage of infected cells was assessed with Image J.

Furthermore, another ten genetically different viral strains were used for comparison of the replication in both PK15^S10–CD163^, PK15^S1–CD163^ cell line using the sample protocol as described above (Table 1). The ten different PRRSV strains grown on PAMs were: 94V360, 07V063, 08VA, 13V091, 13V117, 17V024, Korean17, Su1-Bel, NADC 30, SDSU73.

### Statistical analysis

Infection experiments for the four prototype strains were performed with at least three replicates. Results were compared using a two-way ANOVA test (GraphPad Prism 5) followed by Šidak’s multiple comparisons test. Differences were considered statistically significant at *p* < 0.05. Variance between intracellular and extracellular titers for the two cell lines were analyzed in univariate main effect model with SPSS. Differences were considered statistically significant at *p* < 0.05.

## Results

### Construction of PK15^S10–CD163^ and detection of receptors in the PK15^S1–CD163^ and PK15^S10–CD163^ cell lines by immunofluorescence staining and flow cytometry

**Figure 2:**
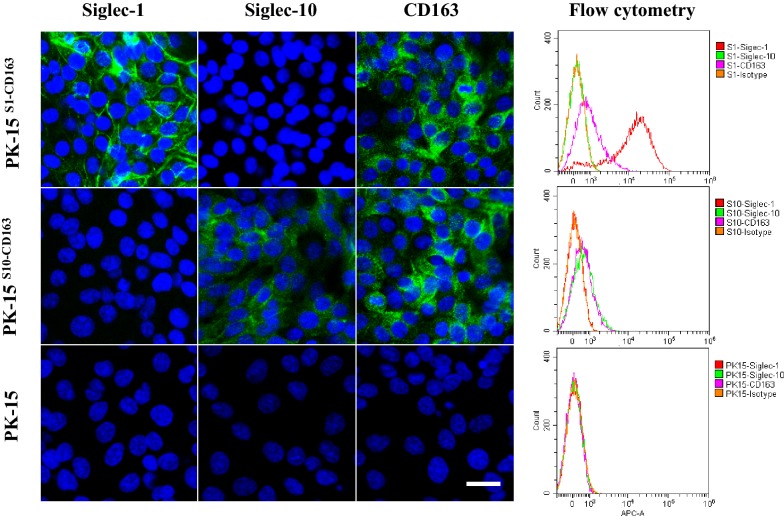
**Detection of receptor expression in PK15, PK15**^**S1–CD163**^ **and PK15**^**S10–CD163**^ **cell lines.** Expression of receptors (Siglec-1, Sigle-10 and CD163) in PK15, PK15^S1–CD163^ and PK15^S10–CD163^ were analyzed by confocal microscopy (left) and flow cytometry (right). Scale bar: 25 µm.

PK15 cells, stably expressing Siglec-10 (PK15^S10^) were produced as previously described [18]. Then, PK15^S10^ cells were transfected with CD163 and screened with zeocin and followed by Facs sorting for selection of double receptors positive cell clones as indicated in Figure 1. The presence of Siglec-10 and CD163 was confirmed by immunofluorescence staining (Figure 1). The cells maintained a stable expression of Siglec-10 and CD163 for at least 15 passages. PK15^S1–CD163^ cells were previously produced by Delrue et al. [10]. Expression of Siglec-1, Siglec-10 and CD163 was analyzed in PK15^S1–CD163^ and PK15^S10–CD163^ cells with immunofluorescence staining and flow cytometry. As shown in Figure 2, the PK15^S1–CD163^ cells were positive for Siglec-1 and CD163, but negative for Siglec-10. PK15^S10–CD163^ cells on the other hand were positive for Siglec-10 and CD163, but negative for Siglec-1. PK15 cells were negative for all of the three receptors (Figure 2).

### Attachment, internalization, disassembly and infection of PK15^S1–CD163^ and PK15^S10–CD163^ cells with PRRSV

**Figure 3:**
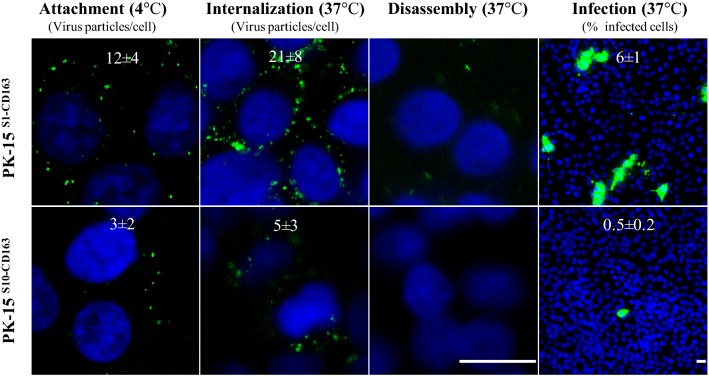
**Attachment, internalization, disassembly and infection of PRRSV in PK15**^**S1–CD163**^ **and PK15**^**S10–CD163**^ **cells.** Cells were inoculated with PAM-grown LV. Then, the different stages of viral replication were visualized with a staining for the nucleocapsid of PRRSV. The experiments were performed in triplicate; the numbers on the images represent mean ± SD of 3 repeats. Scale bar: 25 µm.

Our aim was to compare the attachment, internalization, disassembly and infection of PRRSV in the PK15^S10–CD163^ and PK15^S1–CD163^ cell lines. As shown in Figure 3, virus particles clearly attached to cells of both cell lines and subsequently were internalized into the cells. After internalization, the virus particles were uncoated in order to release the viral genome. Finally, infection occurred (Figure 3). In general, in line with the binding and entry results, the LV strain infected PK15^S1–CD163^ cells better than PK15^S10–CD163^ cells.

### Infection kinetics in PK15^S1–CD163^ and PK15^S10–CD163^ cells upon inoculation with genotype I (LV and Lena) and II (VR2332, MN184) PRRSV reference strains

**Figure 4:**
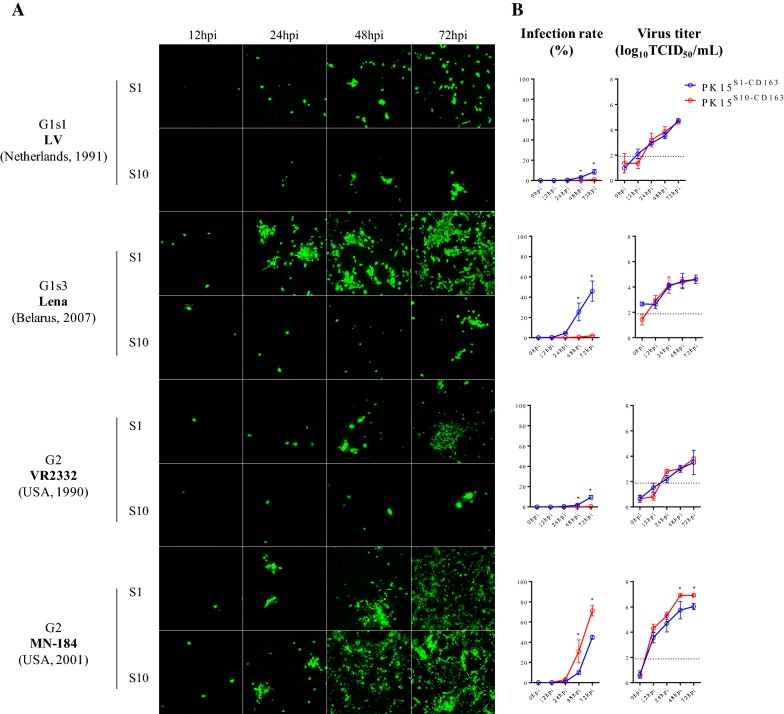
**PRRSV infection kinetics in PK15**^**S1–CD163**^ **and PK15**^**S10–CD163**^ **cells.** Cells were inoculated with PAM-grown PRRSV strains (LV, Lena, VR-2332 and MN-184). After 0, 12, 24, 48 and 72 h of infection, cells were fixed and an immunofluorescence staining was performed (**A**). The number of infected cells was counted and expressed as percentage of infected cells (**B**). In addition, the virus titers in supernatants were determined. The blue line represents the kinetics of the virus infection rate/titers in PK15^S1–CD163^ cells, while the red line represents the kinetics of the virus infection rate/titer in PK15^S10–CD163^ cells. G1s1 = genotype 1 subtype 1; G1s3 = genotype 1 subtype 3; G2 = genotype 2; S1 = PK15^S1–CD163^, S10 = PK15^S10–CD163^. Value of titers and infection proportion presented on the curve graph represent mean ± SD of three experiments. Scale bar: 200 µm.

PK15^S1–CD163^ and PK15^S10–CD163^ cells were inoculated with PAM-grown LV, Lena, MN-184 and VR2332 and at 1, 12, 24, 48 and 72 hpi, cells were fixed and stained by immunofluorescence. Figure 4 shows that the two cell lines are susceptible to all used PRRSV strains and that the infection level increased with time. In general, PAM-grown Lena and MN-184 strains could infect PK15^S1–CD163^ and PK15^S10–CD163^ cells more efficiently than the prototype LV and VR2332 strains (*p* = 0.0081). The percentage of cells infected with LV, Lena, and VR2332 strains was significantly higher in PK15^S1–CD163^ cells than in PK15^S10–CD163^ cell line (*p* < 0.0001). The PAM-grown MN-184 strain showed a significantly higher infection rate in PK15^S10–CD163^ cells (71.2 ± 5.1%) than in PK15^S1–CD163^ cells (44.9 ± 1.8%, *p* < 0.0001). For the LV and VR2332 strains, the virus replication kinetics was 5–12 fold lower compared to the MN-184 and Lena strains; infection was observed only in some cells both for PK15^S1–CD163^ and PK15^S10–CD163^ cells. Interestingly, although there is a higher infection rate for LV, Lena and VR2332 strains in PK15^S1–CD163^ than PK15^S10–CD163^ cells, the virus production in both cell lines was similar. This points towards a higher virus yield per cell for the PK15^S10–CD163^ cells. For the MN-184 strain, a higher virus infection rate and also a higher virus production were found in PK15^S10–CD163^ cells.

### Analysis of Siglec-1, Siglec-10 and CD163 surface and cytoplasm expression in PK15^S1–CD163^ and PK15^S10–CD163^ cells

**Figure 5:**
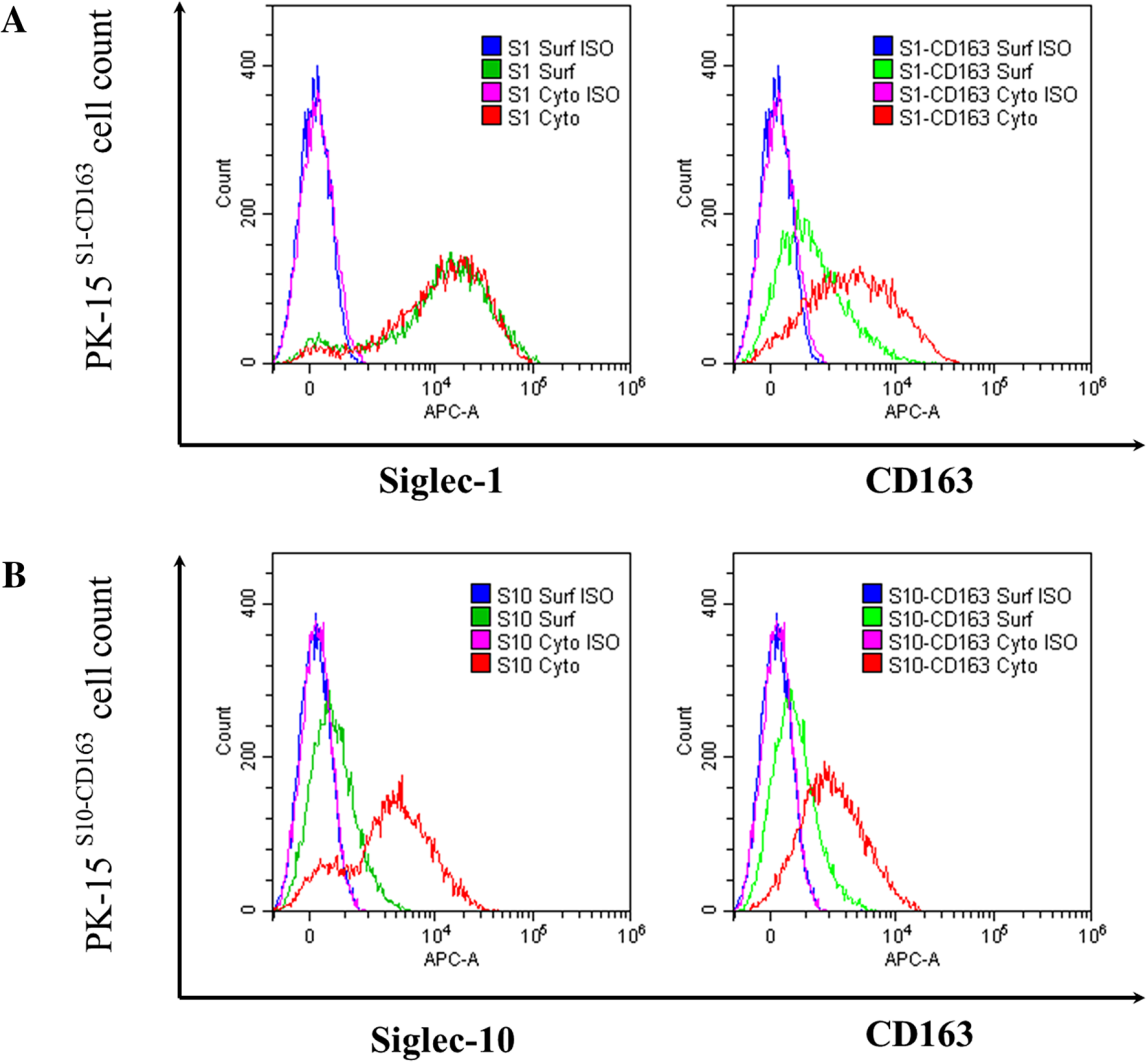
**Flow cytometric analysis of receptor subcellular expression pattern for PK15**^**S1–CD163**^ **and PK15**^**S10–CD163**^ **cell lines.** Siglec-1, Siglec-10 and CD163 expression in the cytoplasm and on the plasma membrane of PK15^S1–CD163^ (**A**) and PK15^S10–CD163^ (**B**) cells were analyzed with flow cytometry. Surf = surface expression; cyto = surface + cytoplasmic expression; ISO = isotype-matched control

Based on the results of the binding and internalization assays, we found that irrespective of the PRRSV strain more virus particles were attached and internalized in the PK15^S1–CD163^ cells. This might explain the higher virus infection rates in PK15^S1–CD163^ cells for the LV, Lena and VR2332 strains. Interestingly, a similar virus production was observed for both cell lines, which might indicate that the virus particles are more anchored to the surface of the PK15^S1–CD163^ cells rather than released in the extracellular environment. As shown Siglec-1^+^ cells showed more binding of virus particles than Siglec-10^+^ cells. We hypothesize that produced virus is anchored at the cell surface, preventing the release of virus particles. To show differences in subcellular expression patterns of Siglec-1 and Siglec-10, we performed a surface and intracellular staining. As shown in Figure 5, Siglec-1 is expressed on the surface of PK15^S1–CD163^ cells to a higher level than Siglec-10 on the surface of PK15^S10–CD163^ cells. In both cell lines, CD163 is expressed on the surface and in the cytoplasma with little difference between the two cell lines.

### Replication kinetics of ten genetically distant PRRSV strains in PK15^S1–CD163^ and PK15^S10–CD163^ cells

**Figure 6:**
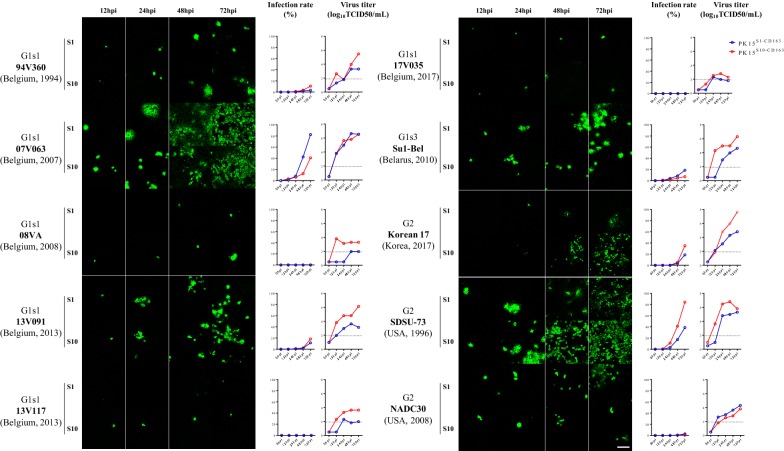
**Comparison of PRRSV infection kinetics in PK15**^**S1–CD163**^ **and PK15**^**S10–CD163**^ **for ten different strains.** Immunofluorescence staining for PRRSV was performed in the two cell lines. After 0, 12, 24, 48 and 72 hpi, cells were fixed and stained for infection. The number of infected cells was counted and expressed as percentage of infected cells. The blue line represents infection rate/virus titers in PK15^S1–CD163^ cells, while the red line represents infection rate/virus titers in PK15^S10–CD163^ cells. G1s1 = genotype 1 subtype 1; G1s3 = genotype 1 subtype 3; G2 = genotype 2; S1 = PK15^S1–CD163^; S10 = PK15^S10–CD163^. Scale bar: 100 µm.

To analyze the possible Siglec preference of different PRRSV strains, we assessed the virus infection/production of additionally 7 genotype 1 subtype 1 strains (G1s1) (94V360, 07V063, 08VA, 13V091, 13V117, 17V035); 1 genotype 1 subtype 3 (G1s3) strains (Su1-Bel) and 3 genotype 2 strains (SDSU-73, NADC30-like and Korea17). Replication kinetics (both virus infection and virus production) were compared in the two cell lines (Figure 6). In general, the PK15^S10–CD163^ cell line exhibited a higher efficiency in virus production per infected cell as compared to PK15^S1–CD163^ (Additional file 1). For G1s1 strains, 07V063 strain preferred PK15^S1–CD163^, whereas the 94V360 and 08VA strains preferred PK15^S10–CD163^. The highly virulent G1s3 strains (Su1-Bel and Lena) showed a strong preference for PK15^S1–CD163^. Similarly, to the genotype 2 strain MN-184 in the previous infection assay, the other two genotype 2 strains, SDSU-73 and Korea17, also showed a higher infection rate in PK15^S10–CD163^ as compared to PK15^S1–CD163^ (84.3% vs 38.7% and 34.93% vs 18.7%, respectively). However, there were no significant differences between PK15^S1–CD163^ and PK15^S10–CD163^ upon infection with VR2332 and NADC30 strains. Some strains (08VA (G1s1), 13V117 (G1s1), VR2332 (G2)) were poor virus producers (<10^4^ TCID_50_/mL), while other strains (07V063 (G1s1), 13V091 (G1s1), Su1-Bel (G1s3), MN-184 (G2), Korean17 (G2) and SDSU-73 (G2)) easily grew up to ≥10^6^ TCID_50_/mL. To confirm that the virus particles are better attached to the PK15^S1–CD163^ cells, leading to a lower efficiency in virus production, both intracellular and extracellular virus titrations were performed for the ten genetically distant virus strains. Interestingly, we found that PK15^S10–CD163^ cells showed much higher extracellular than intracellular virus titers (average titer of 3.41 log_10_TCID_50_/mL vs 2.46 log_10_TCID_50_/mL, *p* = 0.005). However, for PK15^S1–CD163^ cells, the intracellular and extracellular virus titers were comparable (average titer of 2.31log_10_TCID_50_/mL vs 2.45 log_10_TCID_50_/mL, *p* = 0.564) (Additional file 2).

## Discussion

In vivo, PRRSV shows a strict preference for certain macrophage subsets, such as porcine alveolar macrophages (PAM). These primary porcine cells can be used for virus isolation and virus propagation, because they closely mimic the in vivo situation. However, using primary cell lines is expensive and may be a risk for contamination. More importantly, primary cells have a short lifespan and display a batch to batch variation. Apart from PAM, the other cell type known to be permissive to PRRSV is the immortalized monkey kidney cell line MA-104 and its derivatives, such as MARC-145 [11, 22]. This cell line is able to overcome the aforementioned problems and is routinely used for large scale production of PRRSV. However, the use of MARC-145 cell line is limited for virus isolation as some strains (most genotype 1 strains and a certain percentage of genotype 2 strains such as the Korean 17) do not grow in this cell line [23, 24]. In addition, the propagation of PRRSV in MARC-145 cells may lead to genetic changes [25]. The latter feature is used for the development of attenuated vaccines.

Non-permissive cells transiently transfected with CD163 may allow a low level of infection depending on the cell type used for virus replication [26]. Co-expression of Siglec-1 and the scavenger receptor CD163 are needed for an efficient PRRSV infection [27]. In our search to find a cell line that combines the advantages of PAM and MARC-145, our laboratory has already successfully generated a PK15 cell line expressing both Siglec-1 and CD163 (PK15^S1–CD163^). However, we have recently shown that Siglec-1 is not the only entry mediator for PRRSV [18]. As for Siglec-1, Siglec-10 in combination with CD163 is also able to improve PRRSV infection in non-permissive cells. Siglec-10 facilitates attachment and internalization of PRRSV. To assess if the receptor use differs between different PRRSV strains, we constructed a PK15 cell line recombinantly expressing Siglec-10 and CD163 (PK15^S10–CD163^). Expression of both receptors was confirmed with immunofluorescence and flow cytometry.

PRRSV entry in the target cells is initiated via virus-receptor interaction. First, the virus binds to heparin sulphate as well as sialoadhesin, and then, virus is internalized via sialoadhesin through its interaction with the PRRSV GP5-M structural protein complex. Following entry, CD163 coordinates disassembly and the following steps in the virus replication cycle. Previously, we have shown that PK15^S10^ cells were able to bind and internalize PRRSV particles, however, no productive infection could be observed [18]. In this study, CD163 was successfully introduced in the PK15^S10^ cells. The complete replication cycle was analyzed and compared with those in PK15^S1–CD163^ cells. As expected, both cell lines were susceptible for PRRSV infection. Attachment, internalization, disassembly and infection of LV occurred in both cell lines.

The binding and internalization were at a lower level in the PK15^S10–CD163^ cells. We hypothesized that this was due to a different subcellular expression pattern of Siglec-1 and Siglec-10. Siglec-1 is expressed to higher levels on the surface of the cells than Siglec-10. This might partly explain the more efficient binding and internalization in PK15^S1–CD163^ cells. As the CD163 expression is similar in both cell lines, the observed differences can only be attributed to the Siglec localization patterns.

**Figure 7:**
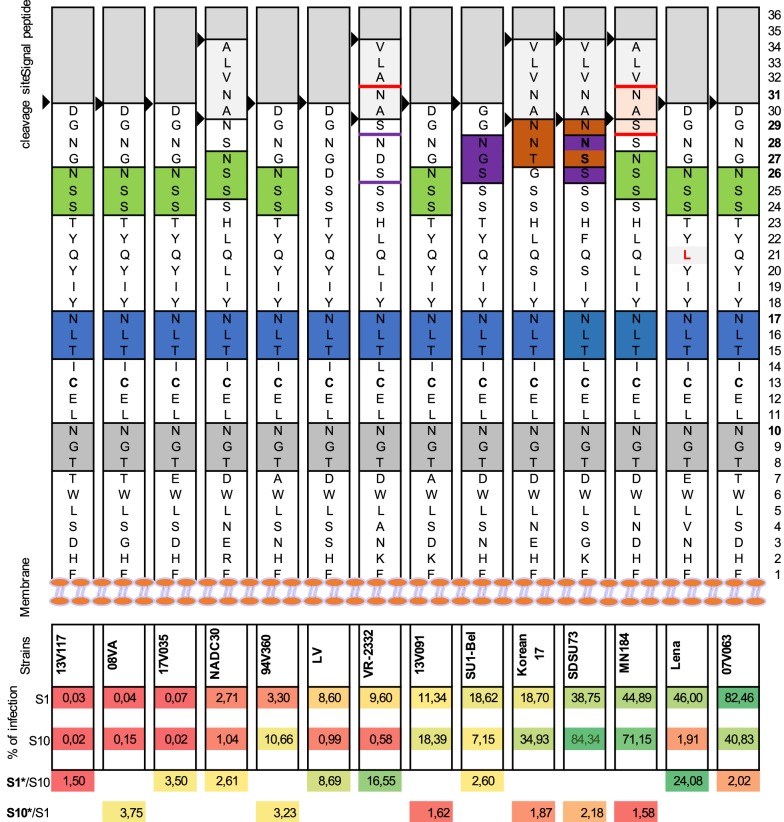
**GP5 ectodomain amino acid and N-glycosylation site analysis for both type 1 and type 2 PRRSV strains.** A graphic representation of an amino acid sequence alignment of the GP5 ectodomain, starting with the 1st amino acid (aa) at the plasma membrane. The potential glycosylation sites were indicated with colored boxes. The first N glycosylation site is N^10^ which is 10 aa away from the membrane (indicated with grey boxes). The second N-glycosylation site N^17^ is colored blue. The third or fourth N-glycosylation sites at N^26^/N^27^ is colored green, N^28^ is colored with purple and at N^31^ is colored with red. Black arrows represent the signal peptide cleavage site for GP5 protein. The virus strains were ranked on the infection rate in Siglec-1 expressing cells. The ranking is represented with a heat map (Red represent a low percentage, yellow represents a medium percentage and green represent high percentage). The preferences for Siglec-1 and Siglec-10 were indicated with an infection ratio of S1/S10 or S10/S1.

In order to determine the efficiency of PRRSV infection and production in PK15^S10–CD163^ compared to PK15^S1–CD163^ cells, a PRRSV replication kinetic experiment with 14 different virus strains was performed. Information on these strains is listed in Table 1. Based on the phylogenetic tree analysis (Additional file 3), the 14 strains used for this study are genetically distant. Significant variability in growth characteristics among genetically distant strains was observed. Some strains [08VA (G1s1), 13V117 (G1s1), VR2332 (G2)] were poor virus producers (<10^4^ TCID_50_/mL), while other strains [07V063 (G1s1), 13V091 (G1s1), Su1-Bel (G1s3), MN-184 (G2), Korean17 (G2) and SDSU-73 (G2)] easily grew up to ≥10^6^ TCID_50_/mL. Interestingly, a previous study from Frydas et al. [16] has shown that the PRRSV strains LV, 08VA and 13V117 were also categorized as poor growers, Lena and VR2332 strains were moderate, while the 07V063, 13V091 and MN-184 strains were strong producers based on virus shedding in respiratory explants [15, 16]. The Korean17 (G2) strain is a recent type 2 strain isolated from a farm with a severe outbreak of PRRS. Our results are consistent with the idea that the virulence is associated with a faster replication in both cell lines. To further analyze the replication of all of the strains, we found that strains LV, 07V063, Sul-Bel, Lena, VR2332 and NADC30 performed better in PK15^S1–CD163^ cells, whereas 94V360, 08VA, 13V091, 13V117, MN-184, SDSU-73 and Korean17 strains preferred PK15^S10–CD163^. Interestingly, the genotype 1 subtype 3 strains (Lena and Su1-Bel) showed a stronger preference for PK15^S1–CD163^ cells, while the highly pathogenic type 2 strains (MN-184, SDSU-73, Korean17) showed a higher affinity to PK15^S10–CD163^ cells. The other two genotype 2 strains, VR2332 and NADC30, showed a slight preference to PK15^S1–CD163^ cells. A previous study [15] revealed that VR2332 mainly infects Sn^+^ cells in the lamina propria of the nasal mucosa, whereas the other genotype 2 strains (MN-184, VN) were also able to infect large number of Sn^-^ cells. This might explain the preference for the cell line observed in this study. No data for the NADC30 strain is available in the ex vivo explant model. However, it was shown that NADC30 strain is relatively less pathogenic compared to MN-184 and SDSU-73 [28]. In return, we can assume that NADC30 might have a relatively more restricted cell tropism compared to the MN-184 and SDSU-73 strains. GP5 is the major glycoprotein of PRRSV. Sugar modification of this viral glycoprotein is important for host–pathogen interactions and signaling recognition. Previously, we have shown that Siglec-1 interacts with the GP5/M heterodimer complex [12]. Siglec-10 functions in a similar way as Siglec-1 in mediating the binding and internalizing of the virus in a sialic acid-dependent manner [18]. Here we further analyzed the amino acid sequence of GP5 for all of the virus strains used in our study with MEGA 6. The first two glycosylation sites (N at position 10 and 17) are fully conserved and are present in all PRRSV strains, demonstrating its crucial role in the binding/internalization process. The third potential N-glycosylation further away from the membrane is more variable (Figure 7). It is highly possible that this variation influences the attachment and internalization due to a siglec preference. The majority of type 2 strains (MN-184, SDSU-73 and Korean 17) carry around 3–4 glycosylation sites (N^10^, N^17^, N^27^/N^28^/N^29^, N^31^) and show a preference for Siglec-10. LV, VR2332 and Lena show a highly preference for Siglec-1. LV has only two glycosylation sites (N^10^, N^17^). Although VR2332 has four potential N-glycosylation sites, only 2 or exceptionally 3N-glycosylation sites are used in nature (N^10^, N^17^, ± N^28^) [29, 30], with a poor glycosylation efficiency of the NDS sequon for N^28^. What Lena concerns, a non-conserved mutation 42Q (polar uncharged) to 42L (hydrophobic) between N^17^ and N^26^ glycosylation site was observed, which changes from an amino acid with an uncharged side chain into a hydrophobic side chain, which might have an impact on the glycosylation modification. However, whether the differences in N-glycosylation sites in the ectodomain of GP5 result in different glycosylation patterns that are related to the preference in usage of Siglecs needs further investigations.

The production of virus by infected cells is an essential process for the spread of viral diseases. Enveloped viruses can spread via two distinct routes, either through the cell-free aqueous environment or by direct cell–cell contact. In the present study, we found that the PK15^S10–CD163^ cell line exhibited a higher efficiency in virus release in the extracellular environment than the PK15^S1–CD163^ cell line (Additional file 1). The prototype strains (LV, Lena, VR2332) showed a much higher infection rate in the PK15^S1–CD163^ cell line, but the virus titers in the supernatant were comparable in both cell lines. Two possible mechanisms may be forwarded for this phenomenon. First, virus might use more cell-to-cell transmission in PK15^S1–CD163^ cells as compared to PK15^S10–CD163^ cells giving rise to a higher infection rate but lower virus release. Second, based on the surface and cytoplasm staining, Siglec-1 is more abundantly expressed on the surface of the cells providing more efficient binding and internalization, while Siglec-10 is mostly expressed in the cytoplasm. This might increase the chance that upon release from the cells, the virus particles bind back to the surface of the PK15^S1–CD163^ cells. This hypothesis is supported by the observation that the extracellular titers were higher for the PK15^S10–CD163^ cell line, while the intra- and extracellular virus titers are more or less the same for the PK15^S1–CD163^ cell line (Additional file 1).

In summary, the observation that all tested virus strains replicate well in both PK15^S1–CD163^ and PK15^S10–CD163^ cells suggesting that these cells might be useful for virus isolation. Today MARC-145 is the mostly used cell line for virus isolation, however, not all PRRSV strains grow on MARC-145 cells [11, 24]. PAM has a much higher efficiency in virus isolation, but it is more variable and more difficult for routine maintenance. The two cell lines that we constructed could be an optimal replacement for both MARC-145 and PAM. In addition, the PK15^S10–CD163^ cell line is more efficient in virus production, which is important for the production of inactivated vaccines. Finally, the observed variability in growth characteristics of all the virus strains tested in both cell lines might be connected with the outcome of a PRRSV infection and might be partially explained by the receptor usage.

## Additional files

**Additional file 1.** **Comparison of virus production per infected cell in PK-15**^**S1–CD163**^ **and PK15**^**S10–CD163**^ **cells**. Blue bars represent the virus production per infected cells of PK-15^S1–CD163^. Red bars represent the virus production per infected cells of PK-15^S10–CD163^.

**Additional file 2.** **Kinetics of intracellular and extracellular virus titers for 10 strains propagated in PK-15**^**S1–CD163**^ **and PK15**^**S10–CD163**^ **cells.** Ten genetically distant PRRSV strains were inoculated in two cell lines. Cells and supernatants were collected separately for titration of extra- and intracellular virus. The blue solid (extracellular) and blue dashed lines (intracellular) represent the titration results for PK-15^S1–CD163^. The red solid (extracellular) and red dashed lines (intracellular) represent the titration results for PK-15^S10–CD163^ cells.

**Additional file 3.** **Phylogenetic tree of virus isolates used in the present study.** A molecular phylogenetic analysis of the full genome nucleotide sequences was constructed using the Neighbor-Joining method. Phylogenetic relationships were estimated using the Clustal Omega method. The optimal tree is drawn to scale. Numbers indicate bootstrap values of 100 replicates. Strain nomenclature is as follows: GenBank accession number/Name of the isolate. Filled circles represent the strains used in the present study.

## Abbreviations

D-MEM: Dulbecco’s Modified Eagle Medium
FBS: fetal bovine serum
FITC: fluorescein isothiocyanate
G1s1: genotype 1 subtype 1
G1s2: genotype 1 subtype 3
G2: genotype 2
LV: Lelystad virus
MAG: myelin-associated glycoprotein
MEM: Modified Eagle Medium
MOI: multiplicity of infection
PAM: porcine alveolar macrophages
PBS: phosphate-buffered saline
pCD163: porcine cluster of differentiation 163
PF: paraformaldehyde
PK-15: porcine kidney cells
pSn: porcine sialoadhesin
PRRSV: porcine reproductive and respiratory syndrome virus
Siglec: sialic acid-binding immunoglobulin-type lectins
