# Supplementary figures and images for: Preferential use of Siglec-1 or Siglec-10 by type 1 and type 2 PRRSV strains to infect PK15S1–CD163 and PK15S10–CD163 cells
Source: Vet Res. 2018 Jul 18;49:67. doi: 10.1186/s13567-018-0569-z (PMC6052533; doi:10.1186/s13567-018-0569-z)

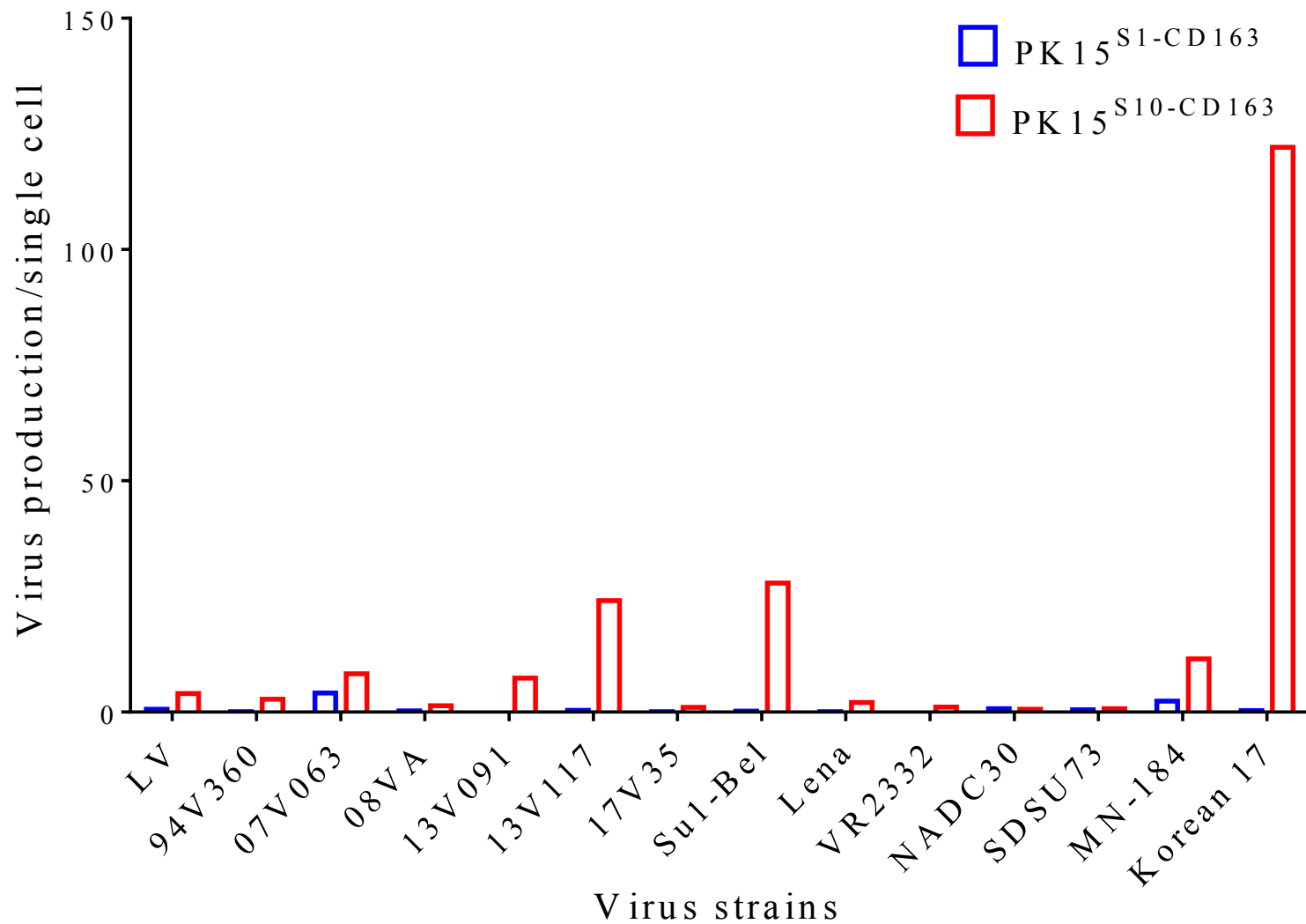

Supplement: Supplementary file 1 — Additional file 1. Comparison of virus production per infected cell in PK-15S1–CD163 and PK15S10–CD163 cells. Blue bars represent the virus production per infected cells of PK-15S1–CD163. Red bars represent the virus production per infected cells of PK-15S10–CD163. [file 13567_2018_569_MOESM1_ESM.pdf]

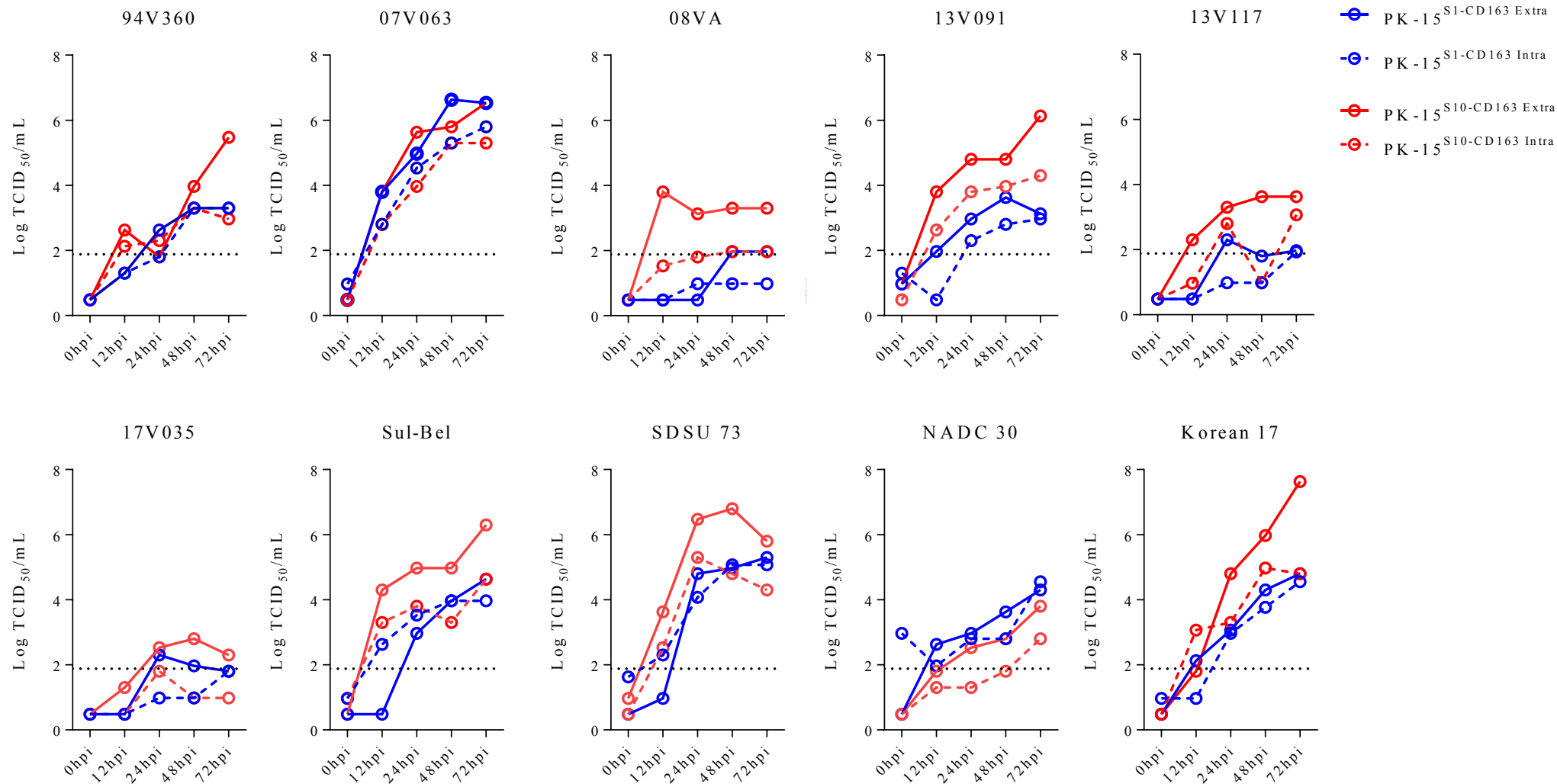

Supplement: Supplementary file 2 — Additional file 2. Kinetics of intracellular and extracellular virus titers for 10 strains propagated in PK-15S1–CD163 and PK15S10–CD163 cells. Ten genetically distant PRRSV strains were inoculated in two cell lines. Cells and supernatants were collected separately for titration of extra- and intracellular virus. The blue solid (extracellular) and blue dashed lines (intracellular) represent the titration results for PK-15S1–CD163. The red solid (extracellular) and red dashed lines (intracellular) represent the titration results for PK-15S10–CD163 cells. [file 13567_2018_569_MOESM2_ESM.pdf]

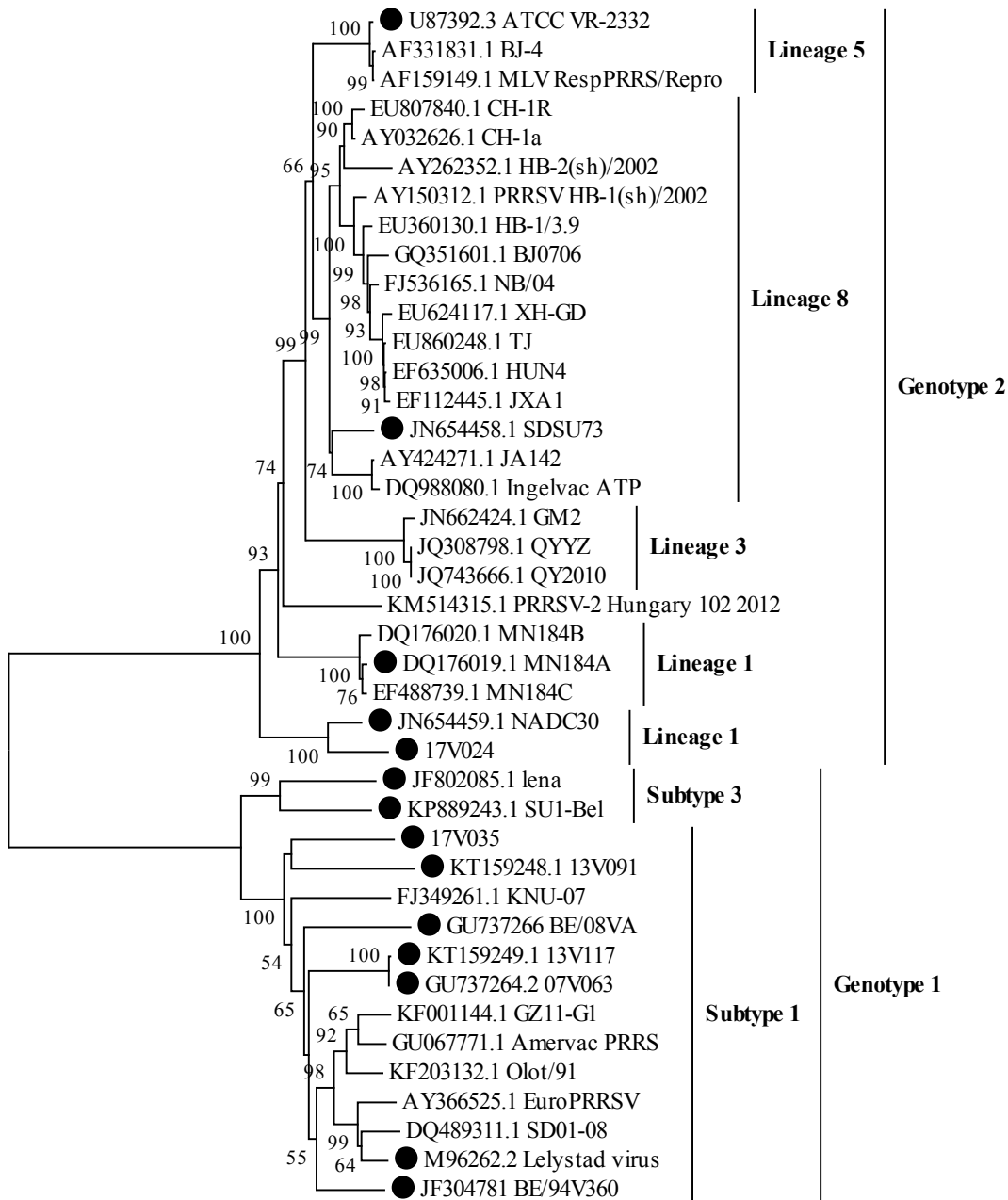

0.05

Supplement: Supplementary file 3 — Additional file 3. Phylogenetic tree of virus isolates used in the present study. A molecular phylogenetic analysis of the full genome nucleotide sequences was constructed using the Neighbor-Joining method. Phylogenetic relationships were estimated using the Clustal Omega method. The optimal tree is drawn to scale. Numbers indicate bootstrap values of 100 replicates. Strain nomenclature is as follows: GenBank accession number/Name of the isolate. Filled circles represent the strains used in the present study. [file 13567_2018_569_MOESM3_ESM.pdf]
